# U.S. state-level variation in caregiver-reported provider recommendation of HPV vaccination to 13-to-17-year-old adolescents

**DOI:** 10.1007/s10552-026-02283-5

**Published:** 2026-11-12

**Authors:** Chinenye Lynette Ejezie, Chengcheng Zhang, Nada Eldawy, Ming Li, Paula Cuccaro

**Affiliations:** 1 Towson University, 8000 York Road, Towson, MD 21252 USA; 2 Charles E. Schmidt College of Medicine, Florida Atlantic University, Boca Raton, FL 33431 USA; 3 UTHealth Houston School of Public Health, 1930 Old Spanish Trail, Houston, TX 77054 USA

**Keywords:** Human papillomavirus vaccine, state-level differences, National Immunization Survey–Teen, United States, provider recommendation, adolescents

## Abstract

**Introduction:**

Although regional differences in provider recommendation of human papillomavirus (HPV) vaccine have been documented, limited research has examined variation at the state level. Therefore, this study examined state-level differences in caregiver-reported health care provider recommendation of HPV vaccines among 13-to-17-year-old adolescents in the United States.

**Method:**

Using a cross-sectional design to examine data from the National Immunization Survey–Teen (2023), multivariable logistic regression was used to model state-specific variations in provider recommendation of HPV vaccine. Proportion estimation was used to examine state-specific prevalence.

**Result:**

Provider recommendation varied considerably across states. For example, the odds of reporting a recommendation were lower among caregivers of adolescents in Arkansas (adjusted odds ratio [AOR] = 0.56, 95% confidence interval [CI] = 0.33 to 0.96), Georgia (AOR = 0.49, 95% CI = 0.26 to 0.93), and Mississippi (AOR = 0.42, 95% CI = 0.24 to 0.74), and higher among caregivers in New Hampshire (AOR = 2.40, 95% CI = 1.23 to 4.68) compared with adolescents in California. Greater heterogeneity was observed in the South compared with other regions. For example, the prevalence of not receiving a recommendation was 16% in Maryland and 41% in Mississippi.

**Conclusion:**

The findings underscore the need to improve HPV vaccine recommendation in some states in the United States. In particular, states with low rates should be targeted for focused interventions.

**Supplementary Information:**

The online version contains supplementary material available at https://doi.org/10.1007/s10552-026-02283-5.

## Introduction

Despite a wealth of interventions to improve human papillomavirus (HPV) vaccine-related communication [1–4] and strong evidence showing that provider recommendations improve uptake [5–7], HPV vaccine recommendation among adolescents in the South Census region of the United States remains low [8, 9]. In a study conducted to examine regional differences in HPV vaccine recommendations among 13-to-17-year-old adolescents, Ejezie et al. [8] found that provider recommendation of HPV vaccination in the South Census region lagged behind the Midwest and Northeast regions. While this information is useful for experts developing programs to address low HPV vaccine recommendation among adolescents, the breakdown according to states remains unclear. Analyzing recommendations by region may mask important differences between individual states, as lower-performing states may be offset by higher-performing states within the same region. Examining recommendations at the state level provides a more granular assessment, allowing for the identification of states that are performing well as well as those that may be lagging behind. This approach can help highlight state-level disparities and identify areas where targeted efforts may be needed.

Adolescents aged 13–17 years represent a particularly important population for examining provider recommendations because they remain eligible for routine and catch-up HPV vaccination [10]. Understanding patterns of provider recommendation within this age group and across states can inform strategies to strengthen vaccination recommendation practices, improve HPV vaccine uptake, and ultimately contribute to the long-term reduction of HPV-related cancers. Therefore, this study was conducted to examine caregiver-reported provider recommendation of HPV vaccination among adolescents (13–17 years old) across the different U.S. states and territories.

## Method

This cross-sectional study employed data from the National Immunization Survey–Teen (NIS-Teen), a population-based phone survey collecting vaccination information for adolescents aged 13–17 years in the United States [11]. The survey aims to collect vaccination information for adolescents aged 13–17 years across all 50 U.S. states, the District of Columbia, and selected U.S. territories. Caregivers provide information regarding their adolescent’s vaccination history and related characteristics. This study focused on adolescents residing in the 50 states and the District of Columbia. The analysis examined whether caregivers reported receiving a health care provider recommendation for HPV vaccination, regardless of the adolescent’s HPV vaccination status. Caregivers were defined as individuals, including biological or adoptive parents, guardians, or other adults, who were responsible for the care of the adolescent. Survey respondents with missing data on provider recommendation, those who answered “Don’t know” or “Refused” to the question about provider recommendation for HPV vaccination, individuals with unknown poverty status, and those without provider-verified data were excluded from the analysis, resulting in a final analytic sample of 14,820. More information about the methods for the NIS-Teen, including data collection and weights generation, can be found elsewhere [11]. The dependent variable was a parent’s or caregiver’s report of whether they have ever received a HPV vaccine recommendation from a health care provider. This was operationalized as a binary variable with only “yes” and “no” retained for analysis. Covariates identified from previous research as being related to the receipt of a HPV vaccine recommendation included sex, age, race/ethnicity, insurance, and poverty. All covariates were operationalized as categorical variables. This study adhered to the STROBE reporting guidelines for cross-sectional studies and was exempt from the institutional review board at Towson University because the data are deidentified and publicly available.

### Statistical analysis

Prevalence estimates and multivariable logistic regression analyses were conducted in Stata/IC version 18 (StataCorp). All analyses accounted for the complex survey design using appropriate strata, primary sampling units, and weights. Proportion estimation was performed to examine state-specific prevalence of provider recommendation for HPV vaccine. Following the prevalence analysis, logistic regression models were used to estimate adjusted odds ratios (ORs) and 95% confidence intervals (CIs) for provider recommendation across states. The logistic regression model adjusted for sex, age, race/ethnicity, insurance status, and household poverty level. California was selected as the reference state because it has the largest population among U.S. states [12] and, consequently, is expected to have the largest number of caregivers represented in the study population. Analyses included only participants with complete information for the outcome and variables used in each analysis. Two-sided *p* values of up to 0.05 were considered statistically significant.

## Results

**Fig. 1:**
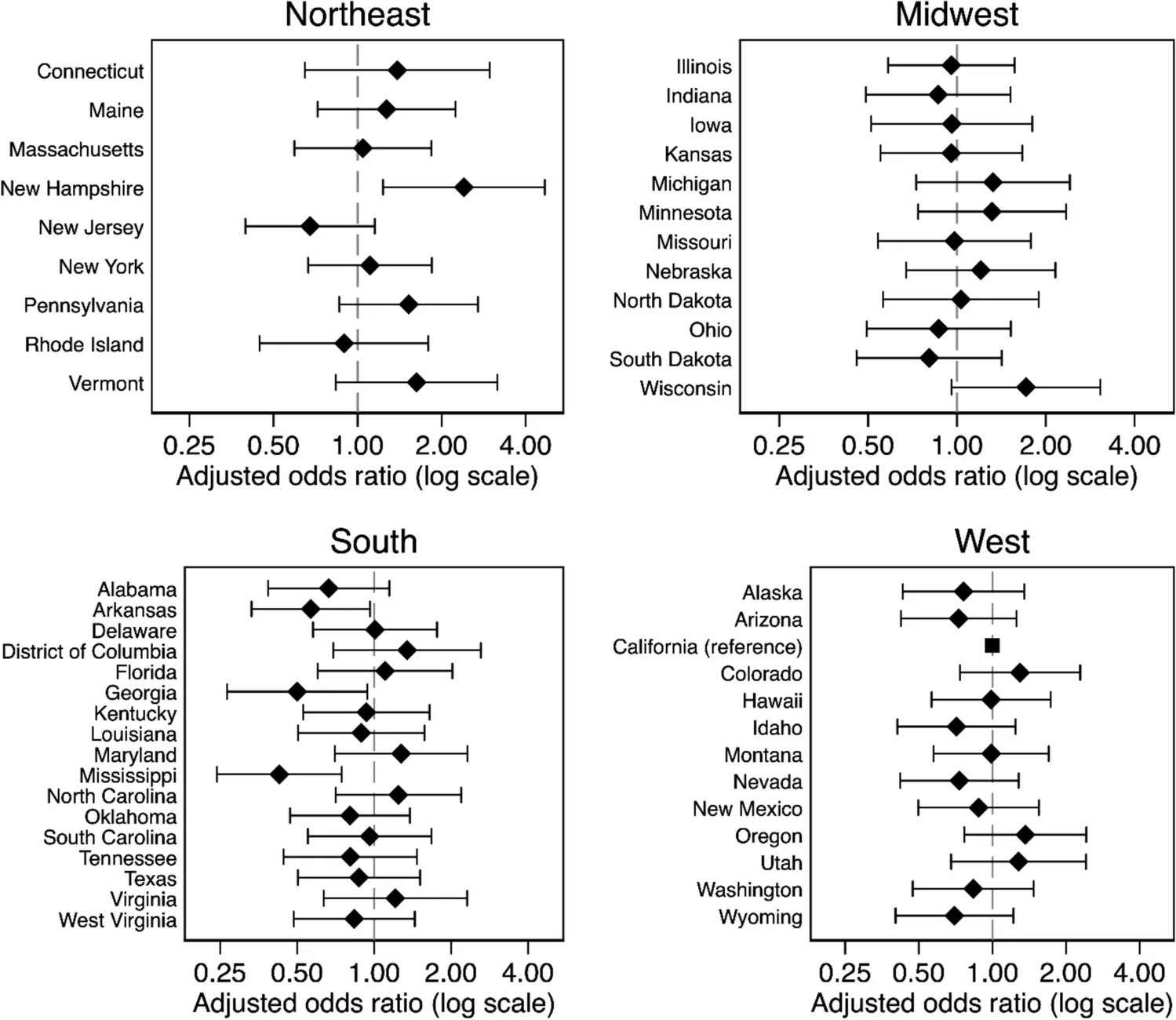
Odds of reporting receipt of a provider recommendation according to state among 13–17-year-olds in overall sample, National Immunization Survey-Teen (2023; *n* = 14,820)

Substantial state-level variation in provider recommendation was observed. Specifically, caregivers of adolescents in several states particularly in the South Census region, had lower odds of reporting receipt of a provider recommendation compared with the reference state (California). For example, the odds of reporting receipt of a provider recommendation for HPV vaccination were significantly lower for caregivers of adolescents in Arkansas (adjusted odds ratio [AOR] = 0.56, 95% confidence interval [CI] = 0.33 to 0.96), Georgia (AOR = 0.49, 95% CI = 0.26 to 0.93), and Mississippi (AOR = 0.42, 95% CI = 0.24 to 0.74) compared with adolescents in California. In contrast, caregivers of adolescents in New Hampshire had higher odds (AOR = 2.40, 95% CI = 1.23 to 4.68) of reporting a health care provider recommendation compared with caregivers of adolescents in California. Within regions, provider recommendation varied considerably across Southern states, with greater heterogeneity observed in this region than in the Northeast and Midwest. For example, most state-level findings did not differ significantly from those observed in California. However, in the South, caregivers in Georgia and Mississippi had lower odds of reporting a health care provider recommendation (AOR = 0.49, 95% CI = 0.26 to 0.93; AOR = 0.42, 95% CI = 0.24 to 0.74 respectively) compared with caregivers in California, whereas the findings for some states including Maryland and Virginia were not statistically significant. Figure 1; Table 1. Most covariates included in the analysis (sex, age, insurance status, and poverty status) demonstrated associations with provider recommendation Supplemental Table 1.

**Table 1:** Prevalence of no provider recommendation and odds of reporting receipt of a provider recommendation according to state among 13–17-year-olds in overall sample, National Immunization Survey-Teen (2023; *n* = 14,820)

Region and state		Prevalence of no HPV vaccine recommendation		Odds of receiving HPV vaccine recommendation	Region and state		Prevalence of no HPV vaccine recommendation		Odds of receiving HPV vaccine recommendation
Region	State abbreviation	No recommendatio*n* (%)	95% CI	Adjusted OR (95% CI)^a^	Region	State abbreviation	No recommendatio*n* (%)	95% CI	Adjusted OR (95% CI)^a^
Midwest	IA	23	18.5–28.0	0.96 (0.51–1.79)	South	GA	32	25.9–38.0	0.49 (0.26–0.93)
Midwest	IL	24	20.8–26.2	0.95 (0.58–1.56)	South	KY	29	24.7–33.7	0.93 (0.52–1.64)
Midwest	IN	28	23.2–32.0	0.86 (0.49–1.51)	South	LA	28	24.2–32.2	0.89 (0.50–1.57)
Midwest	KS	26	21.9–30.9	0.95 (0.55–1.66)	South	MD	16	12.2–19.0	1.27 (0.70–2.31)
Midwest	MI	24	19.4–29.5	1.32 (0.72–2.41)	South	MS	41	36.2–46.3	0.42 (0.24–0.74)
Midwest	MN	18	14.8–21.8	1.31 (0.73–2.34)	South	NC	28	24.2–32.7	1.24 (0.70–2.19)
Midwest	MO	29	23.8–33.4	0.98 (0.54–1.77)	South	OK	32	27.6–36.4	0.80 (0.46–1.37)
Midwest	ND	19	14.8–23.1	1.03 (0.56–1.89)	South	SC	27	23.3–31.2	0.96 (0.55–1.67)
Midwest	NE	23	18.6–27.0	1.20 (0.67–2.15)	South	TN	27	21.7–32.8	0.80 (0.44–1.47)
Midwest	OH	27	22.7–31.9	0.86 (0.49–1.52)	South	TX	30	25.8–34.2	0.87 (0.50–1.51)
Midwest	SD	23	18.5–26.6	0.80 (0.45–1.41)	South	VA	25	19.9–29.6	1.21 (0.63–2.31)
Midwest	WI	20	16.5–23.4	1.71 (0.95–3.06)	South	WV	26	22.6–30.1	0.83 (0.48–1.44)
Northeast	CT	20	14.3–25.6	1.38 (0.64–2.97)	West	AK	33	27.8–38.2	0.76 (0.43–1.35)
Northeast	MA	18	13.9–21.6	1.04 (0.59–1.83)	West	AZ	32	27.8–36.7	0.72 (0.42–1.25)
Northeast	ME	16	13.0–19.2	1.26 (0.71–2.24)	West	CA	29	24.1–33.0	Ref^b^
Northeast	NH	12	9.0–15.1	2.40 (1.23–4.68)	West	CO	19	15.3–22.1	1.29 (0.73–2.28)
Northeast	NJ	27	23.4–31.2	0.67 (0.39–1.15)	West	HI	24	19.7–27.7	0.98 (0.56–1.72)
Northeast	NY	22	19.2–25.3	1.10 (0.66–1.84)	West	ID	33	27.7–37.7	0.71 (0.40–1.24)
Northeast	PA	19	15.2–22.1	1.52 (0.86–2.70)	West	MT	30	25.7–33.9	0.98 (0.57–1.69)
Northeast	RI	22	15.7–27.7	0.89 (0.44–1.79)	West	NM	27	22.8–32.0	0.87 (0.49–1.55)
Northeast	VT	11	6.9–14.6	1.62 (0.83–3.16)	West	NV	33	28.0–37.1	0.73 (0.42–1.28)
South	AL	29	25.2–33.3	0.66 (0.38–1.14)	West	OR	21	17.1–24.9	1.36 (0.76–2.42)
South	AR	32	27.6–36.7	0.56 (0.33–0.96)	West	UT	22	17.3–26.9	1.28 (0.67–2.41)
South	DC	17	13.5–20.9	1.34 (0.69–2.61)	West	WA	26	20.8–31.3	0.83 (0.47–1.47)
South	DE	19	15.6–22.8	1.00 (0.57–1.76)	West	WY	30	25.4–34.1	0.70 (0.40–1.21)
South	FL	24	19.3–28.4	1.10 (0.60–2.02)					

^a^Model was adjusted for sex, age, race and ethnicity, insurance, and poverty

^b^California was the reference state

Findings following the prevalence analysis show state level differences and that individuals in Southern states have a lower probability of reporting receipt of a HPV vaccination recommendation. For example, the prevalence of reporting not receiving a provider recommendation in Mississippi was 41%. The findings also show substantial variation across Southern states. For example, the prevalence of reporting not receiving a health care provider recommendation was 16% in Maryland compared with 41% in Mississippi. Additionally, the results show that some Northeastern and Western states stand out with higher probabilities of reporting receipt of a recommendation. For example, only 12% of respondents in New Hampshire and 19% in Colorado reported not receiving a provider recommendation for HPV vaccination Table 1.

## Discussion

This study is the first to examine caregiver-reported health care provider recommendation of HPV vaccination among adolescents across U.S. states using the 2023 NIS-Teen data. The findings highlight important state-level differences in caregiver-reported provider recommendations of HPV vaccination, underscoring variation in adolescent HPV vaccination promotion across the United States. Understanding state-level variation may help identify local patterns that are masked by broader regional analyses and inform more targeted public health policies and interventions.

Some Southern states, including Mississippi, Georgia, and Arkansas, underperformed relative to California, the reference state. This finding may, to some extent, explain the lower vaccination rates observed in these states [13], as health care provider recommendation is a strong driver of vaccine uptake [5–7]. The lower rates of HPV vaccination recommendation in the South may be partially explained by barriers that health care providers in some states in this region encounter when recommending the vaccine. These barriers include lack of training, time constraints, and lack of effective tools and information to give patients [14]. The prevalence and impact of these barriers likely vary across Southern states, which may contribute to differences in provider recommendation practices and vaccination coverage. Additional research is needed to better understand the state-specific factors underlying these disparities and to inform the development and evaluation of targeted interventions aimed at improving provider recommendations.

Interestingly, substantial heterogeneity was observed within the South. Several states in this traditionally low-performing region demonstrated high rates of provider recommendation, highlighting the limitations of relying solely on regional analyses. For example, Maryland had a relatively low prevalence of caregivers of adolescents reporting not receiving a provider recommendation for HPV vaccination, suggesting that regional averages may mask important state-level differences. By identifying states with the lowest and highest recommendation rates within a region, the findings help determine which states contribute to the region’s overall low rates and pinpoints where additional public health efforts are most needed to improve the recommendation of HPV vaccine. Further research using longitudinal data is needed to confirm this finding.

A limitation of this study is that the question used to measure provider recommendation (“Has a doctor or other health care professional ever recommended that [teen name] receive HPV shots?”) was not time-bound. Also, it was subject to recall bias. Nevertheless, this question is useful in examining provider recommendation across states in the United States and therefore is applicable to our research. Another limitation is that parental or guardian education was not included as a covariate in the analysis. This factor may be important in studies of vaccine recommendation because education level could influence how caregivers understand, recall, or interpret information provided by health care providers. Nevertheless, we included other strong and established covariates that are known to be associated with HPV vaccine recommendation. Another limitation is that differences across states in missing, “don’t know,” or “refused” responses to the health care provider recommendation question were not specifically examined. Although all missing data were excluded from the analysis, variation in these response patterns could affect the comparability of state-level estimates and may introduce response bias if adolescents with nonresponse differed from those who provided responses. Future studies should examine these response patterns to better understand their potential impact on findings. Strengths include the large sample size and nationally representative data.

## Conclusion

This study highlights important state-level variation in the recommendation of HPV vaccine, revealing differences among countries both within and across regions. The presence of both high and low HPV vaccine recommendation rates within regions underscores the need for further research to better understand these differences.

## Supplementary Information

Below is the link to the electronic supplementary material.

Supplementary file1 (DOCX 28 KB)

## Data Availability

Data available upon request.
