# Supplementary material for: U.S. state-level variation in caregiver-reported provider recommendation of HPV vaccination to 13-to-17-year-old adolescents
Source: Cancer Causes Control. 2026 Nov 12;37(12):196. doi: 10.1007/s10552-026-02283-5 (PMC13759712; doi:10.1007/s10552-026-02283-5)
Supplement: Supplementary file 1 [file 10552_2026_2283_MOESM1_ESM.docx]

Supplemental Table 1: Covariates associated with the odds of receiving a provider recommendation according to state among 13–17-year-olds in overall sample, National Immunization Survey-Teen (2023; n=14,820).

| **Variable / Category** | **Adjusted OR (95% CI) – Provider Recommendation** |
| --- | --- |
| **Sex** |  |
| Male | Ref |
| Female | 1.83 (1.56–2.15) |
| **Age (years)** |  |
| 13 | Ref |
| 14 | 1.17 (0.92–1.48) |
| 15 | 1.25 (0.99–1.59) |
| 16 | 1.45 (1.13–1.85) |
| 17 | 1.82 (1.42–2.32) |
| **Race/Ethnicity** |  |
| Hispanic | Ref |
| Non-Hispanic White | 1.17 (0.94–1.46) |
| Non-Hispanic Black | 1.37 (0.99–1.89) |
| Non-Hispanic Other | 1.16 (0.88–1.53) |
| **Insurance** |  |
| Private insurance | Ref |
| Uninsured | 0.26 (0.11–0.59) |
| Any Medicaid | 1.11 (0.77–1.62) |
| Other Insurance | 0.56 (0.31–0.99) |
| **Poverty** |  |
| Above > $75,000 | Ref |
| Below Poverty | 0.41 (0.32–0.50) |
| Poverty – Above ≤ $75,000 | 0.58 (0.48–0.70) |
